# Clozapine modifies the differentiation program of human adipocytes inducing browning

**DOI:** 10.1038/tp.2016.230

**Published:** 2016-11-29

**Authors:** E Kristóf, Q-M Doan-Xuan, A K Sárvári, Á Klusóczki, P Fischer-Posovszky, M Wabitsch, Z Bacso, P Bai, Z Balajthy, L Fésüs

**Affiliations:** 1 Department of Biochemistry and Molecular Biology, University of Debrecen, Debrecen, Hungary; 2 Department of Biophysics and Cell Biology, University of Debrecen, Debrecen, Hungary; 3 Division of Pediatric Endocrinology and Diabetes, University Medical Center Ulm, Ulm, Germany; 4 MTA-DE Lendület Laboratory of Cellular Metabolism, Debrecen, Hungary; 5 Research Center for Molecular Medicine, Faculty of Medicine, University of Debrecen, Debrecen, Hungary; 6 Department of Medical Chemistry, University of Debrecen, Debrecen, Hungary; 7 MTA-DE Stem Cells, Apoptosis and Genomics Research Group of the Hungarian Academy of Sciences, Debrecen, Hungary

## Abstract

Administration of second-generation antipsychotic drugs (SGAs) often leads to weight gain and consequent cardio-metabolic side effects. We observed that clozapine but not six other antipsychotic drugs reprogrammed the gene expression pattern of differentiating human adipocytes *ex vivo*, leading to an elevated expression of the browning marker gene *UCP1*, more and smaller lipid droplets and more mitochondrial DNA than in the untreated white adipocytes. Laser scanning cytometry showed that up to 40% of the differentiating single primary and Simpson–Golabi–Behmel syndrome (SGBS) adipocytes had the characteristic morphological features of browning cells. Furthermore, clozapine significantly upregulated *ELOVL3*, *CIDEA*, *CYC1*, *PGC1A* and *TBX1* genes but not *ZIC1* suggesting induction of the beige-like and not the classical brown phenotype. When we tested whether browning induced by clozapine can be explained by its known pharmacological effect of antagonizing serotonin (5HT) receptors, it was found that browning cells expressed 5HT receptors 2A, 1D, 7 and the upregulation of browning markers was diminished in the presence of exogenous 5HT. Undifferentiated progenitors or completely differentiated beige or white adipocytes did not respond to clozapine administration. The clozapine-induced beige cells displayed increased basal and oligomycin-inhibited (proton leak) oxygen consumption, but these cells showed a lower response to cAMP stimulus as compared with control beige adipocytes indicating that they are less capable to respond to natural thermogenic anti-obesity cues. Our data altogether suggest that novel pharmacological stimulation of these masked beige adipocytes can be a future therapeutic target for the treatment of SGA-induced weight gain.

## Introduction

Obesity is one of the major risk factors of metabolic syndrome, coronary heart disease and cancer, which are leading causes of morbidity and mortality today.^1, 2^ Recent studies showed high incidence of metabolically active brown adipose tissue (BAT), which can dissipate energy directly into heat in healthy adult humans and revealed the strong negative correlation between obesity and the amount of BAT.^3, 4, 5^ Energy expenditure of BAT is not exclusively mediated by the *ab ovo*-differentiated classical brown adipocytes.^6^ Beige cells that can be found interspersed in subcutaneous white adipose tissue (WAT) and generated in a process called ‘browning' also has a significant role.^7, 8, 9^ Although several studies suggest that a large proportion of the thermogenic fat depots in adult humans is mostly composed of beige cells, there is only limited information about the origin of beige adipocytes and the regulators of beige adipogenesis in humans.^10, 11, 12^ The available data obtained from mouse models and human samples suggest that the differentiation of beige cells, characterized by the elevated expression of the beige-selective *TBX1* gene,^10^ is mostly induced from a distinct precursor as a result of several stimuli (for example, cold, physical exercise, diet) at least partially regulated by the β-adrenergic signaling pathway.^13, 14^ Beige adipocyte development can be highly enhanced by several neuro-endocrine or paracrine factors (for example, norepinephrine, irisin, atrial natriuretic peptide, bone morphogenic protein-7), which determine a beige potential or thermogenic competency of each individual.^15, 16, 17^ When the thermogenic stimulus subsides, ‘masked' beige cells persist that they, however, have a white adipocyte-like morphology *in vivo*.^14, 18^ Both ‘mature' and ‘masked' beige adipocytes are able to activate *UCP1* expression and their thermogenic capacity in response to a recurring β-adrenergic stimulus.^10, 14, 18^ Thus, the augmentation of beige potential or the identification and subsequent thermogenic induction of ‘masked' beige adipocytes, for example, by stimulating the β3-adrenergic pathway can be a future therapeutic target against obesity and type 2 diabetes mellitus.^19, 20^

The prevalence rate of obesity and its comorbidities is at least two times higher in patients suffering from schizophrenia or other severe mental illnesses compared with the general population.^21, 22^ Moreover, the long-term administration of second-generation antipsychotic drugs (SGAs), especially clozapine, olanzapine, risperidone and quetiapine further increases the incidence of weight gain and metabolic syndrome in patients with severe mental illnesses.^23, 24, 25^ Different mechanisms were proposed, which might underlie the SGA-induced weight gain.^26^ These include the increase of appetite related to the activation of hypothalamic AMP kinase via histamine H1 receptors in rodents,^27^ decrease of insulin sensitivity in rats,^28^ defects in insulin secretion or clearance in dogs^29^ or rats,^30^ altered gut microbiota in rats^31^ and humans,^32^ induced low-grade inflammation in the adipose tissue in rats^33, 34^ and humans^35^ and direct stimulation of adipogenesis in rodents.^36, 37^

Previously, we demonstrated that the long-term SGA administration enhanced the expression of several white and general adipogenic marker genes and pro-inflammatory mediators in differentiating human adipocytes.^35^ Surprisingly, we also observed that the treatment with clozapine but not with other antipsychotic drugs resulted in the elevated expression of the major brown and beige adipocyte marker gene, *UCP1*. Using gene expression measurements, functional analysis of mitochondrial respiration and the recently developed laser scanning cytometry (LSC)-based technique, which could clearly discriminate between the white and brown adipocytes in cell culture conditions,^38^ we found that clozapine modified the differentiation program of human adipocyte precursors, presumably via the inhibition of serotonin (5HT) receptor-mediated signaling, leading to the generation of beige adipocytes with masked and not fully responsive thermogenic potential.

## Materials and methods

### Materials

All the chemicals were from Sigma-Aldrich (Munich, Germany) unless stated otherwise.

### Ethics statement

Human adipose-derived mesenchymal stem cells (hADMSCs) were isolated from subcutaneous abdominal adipose tissue of healthy volunteers (body mass index <29.9) aged 20–65 years who underwent a planned surgical treatment (herniotomy). Written informed consent from all the participants was obtained before the surgical procedure. The study protocol was approved by the Ethics Committee of the University of Debrecen, Hungary (No. 3186-2010/DEOEC RKEB/IKEB). All the experiments were carried out in accordance with the approved ethical guidelines and regulations.

### Isolation of hADMSCs, cell culture, *ex vivo* differentiation induction and treatments

The hADMSCs were isolated and cultivated from the stromal-vascular fraction (SVF) of abdominal subcutaneous fat as described previously.^35, 38^ White^35, 38, 39, 40^ and positive control beige adipocytes^38, 41, 42^ were differentiated from the hADMSCs, or from the Simpson–Golabi–Behmel syndrome (SGBS) preadipocyte cell line^39, 40^ (University Medical Center Ulm, Ulm, Germany) according to the already described protocols. The absence of mycoplasma was checked by polymerase chain reaction (PCR) analysis (PCR Mycoplasma Test Kit I/C, PromoKine, PromoCell France). The antipsychotic drugs were dissolved in dimethyl sulfoxide and differentiating adipocytes were treated every day in the following final concentrations: olanzapine 50 ng ml^−1^, ziprasidone 50 ng ml^−1^, quetiapine 50 ng ml^−1^, aripiprazole 100 ng ml^−1^, haloperidol 10 ng ml^−1^ and risperidone 50 ng ml^−1^.^35^ Clozapine was administered at 100 ng ml^−1^ concentration to the hADMSCs and to the fully differentiated white or brown adipocytes for 12 h (short-term treatment) or on the last 2 and 4 days or during the whole white adipogenic differentiation process (long-term treatment). Where indicated, the cells were treated with 5HT at 10 μm concentration during the whole adipocyte differentiation.^43^ To investigate the response of differentiated adipocytes to thermogenic induction, the cells received a single bolus of dibutyril-cAMP at 500 μm concentration for 4 h.^6^

### RNA and DNA preparation, PCR array and TaqMan real-time PCR

The messenger RNA (mRNA) expressions in response to the treatment with the seven antipsychotic drugs were determined with CAPH09329 Custom Human RT2 Profiler PCR Arrays (SABiosciences, Frederick, MD, USA) as described previously.^35^ See reference 38 for details about TaqMan Real-time PCR. Mitochondrial DNA quantification by Quantitative PCR was carried out as described previously.^38^ Quantitative PCR experiments were repeated at least five times with SVFs from independent healthy donors or with SGBS samples from independent passages.

### Immunofluorescence staining, image acquisition and quantification of *ex vivo* brown adipocyte differentiation

The hADMSCs or SGBS cells were plated on Ibidi eight-well microslides and differentiated as previously described. Vital and immunofluorescence stainings were carried out as described previously.^38^ First- and second-sample scanning was done by iCys Research Imaging Cytometer (iCys, Thorlabs Imaging Systems, Sterling, VA, USA). The images were processed and analyzed (*n=*3, 2000 cells per donor or SGBS sample) by our high-throughput automatic cell-recognition protocol using the iCys companion software (iNovator Application Development Toolkit, CompuCyte Corporation, Westwood, MA, USA) and CellProfiler (The Broad Institute of MIT, MA, USA). See reference ref. 38 for further details about the analysis.

### Immunoblotting

The polyvinylidene fluoride membranes were probed by polyclonal anti-Ucp1 (U6382) and monoclonal anti-Gapdh (Millipore, Billerica, MA, USA, MAB374) antibodies overnight at 4 °C, followed by incubation with horseradish-peroxidase-conjugated species-corresponding secondary antibodies (Covalab, Villeurbanne, France, lab0252 and lab0273) for 1 h at room temperature. Densitometry was carried out using Image J software (*n=*3). See reference ref. 38 for a more detailed description.

### Oxygen consumption

Oxygen consumption was measured using an XF96 oxymeter (Seahorse Biosciences, North Billerica, MA, USA). The hADMSCs were seeded and differentiated in 96-well XF96 assay plates. After recording the baseline OC, the cells received a single bolus of dibutyril-cAMP at 500 μm concentration modeling adrenergic stimulation. Then, stimulated OC was recorded every 30 min. The final reading took place at 5 h post treatment. To exclude that dibutyril-cAMP enhanced lipolysis might provoke artefactual mitochondrial uncoupling, we repeated the OC measurements including 2% BSA in the respiration buffer.^44^ The adipocytes were treated with 5 μm etomoxir or with 2 mm β-guanidinopropionic acid to block beta-oxidation and creatine-driven substrate cycle.^45^ Next, proton leak respiration was determined after adding oligomycin at 2 μm concentration to block ATP synthase activity. As a last step, the cells received a single bolus of Antimycin A at 10 μm concentration for baseline correction. The oxygen consumption rate (OCR) was normalized to protein content and normalized readings were displayed. For statistical analysis (*n=*4), the fold change of OC levels were calculated comparing the basal, cAMP-stimulated and oligomycin-inhibited (both in unstimulated and stimulated cells) OCRs of each sample to the basal OCR of untreated white adipocytes.^38, 42, 46^

### Statistical analysis

The results are expressed as the mean±s.d. for the number of assays indicated. The sample sizes were chosen to ensure adequate statistical power following the practice of similar studies.^35, 38, 40, 42^ The normality of distribution of the data was tested by Kolmogorov–Smirnov test. For multiple comparisons of groups, statistical significance was calculated and evaluated by one-way analysis of variance followed by Tukey *post hoc* test. In comparison of two groups, two-tailed Student's *t*-test was used. The data were analyzed using Prism 6.01 (GraphPad Software, San Diego, CA, USA).

## Results

### Long-term clozapine treatment induces a beige-like gene expression pattern in differentiating adipocytes

The hADMSCs were cultivated from the SVF of abdominal subcutaneous fat^35, 38^ and a previously described differentiation protocol was applied to induce white adipocyte differentiation.^35, 39^ Using this *ex vivo* approach, up to 50% of the treated hADMSCs were able to accumulate large lipid droplets, which could be quantified by LSC in a time-dependent manner.^40^ Primarily, we aimed to investigate the direct effect of seven antipsychotic drugs at doses comparable to their therapeutic plasma concentrations^35, 47, 48, 49, 50^ on the gene expression of the differentiating adipocytes. After a 14 days long white adipogenic differentiation^39^ and parallel daily administration of antipsychotics, whole cell lysates were collected, then mRNA expressions were determined by a custom PCR Array.^35^ We found that clozapine administration resulted in a sixfold upregulation of *UCP1*, the major brown and beige adipocyte marker gene.^51^ None of the used other five SGAs (olanzapine, ziprasidone, quetiapine, aripiprazole and risperidone) and a typical antipsychotic (haloperidol) influenced the expression of *UCP1* (Supplementary Figure 1).

**Figure 1:**
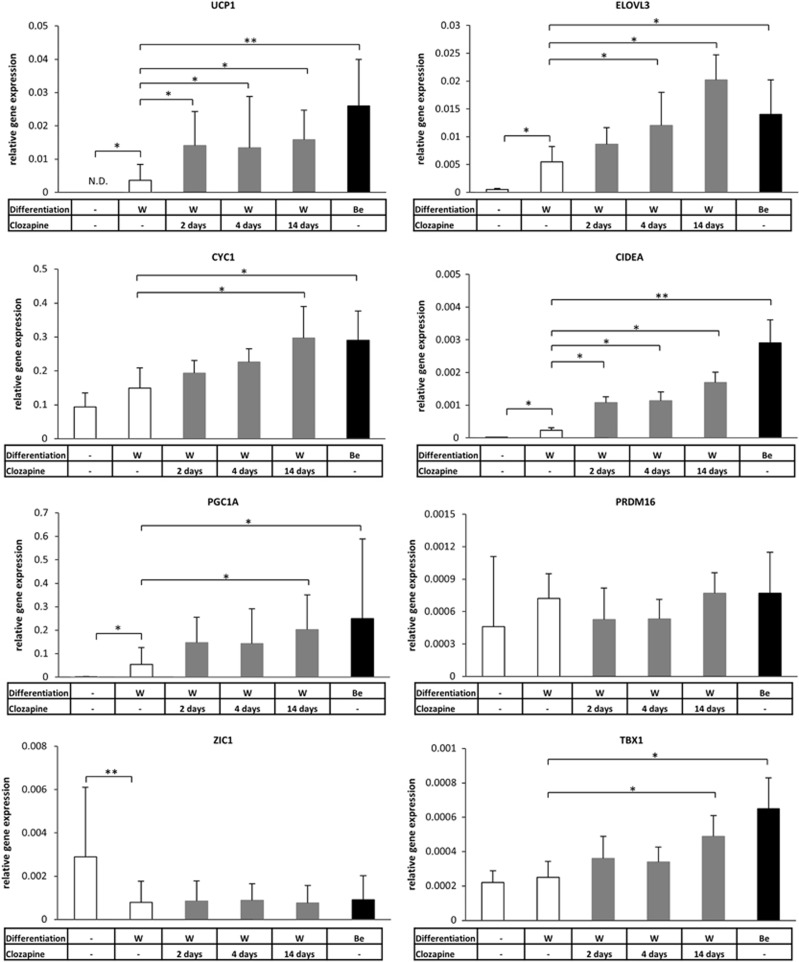
Relative expression of brown and beige adipogenic markers and key transcriptional regulators of brown adipocyte development in primary human adipocytes as a result of clozapine treatment during *ex vivo* white or beige adipocyte differentiation. SVF-derived hADMSCs were differentiated for 2 weeks to white (W) or positive control beige (Be) adipocytes. Clozapine (gray bars) was administered at 100 ng ml^−1^ concentration on the last 2 and 4 days or during the whole white adipogenic differentiation process. The experiment was repeated six times with SVFs from independent healthy donors (Relative gene expression was determined by RT-qPCR, target genes were normalized to *GAPDH*); *P<0.05, **P<0.01. hADMSC, human adipose-derived mesenchymal stem cell; RT-qPCR, quantitative PCR with reverse transcription; SVF, stromal-vascular fraction.

**Figure 2:**
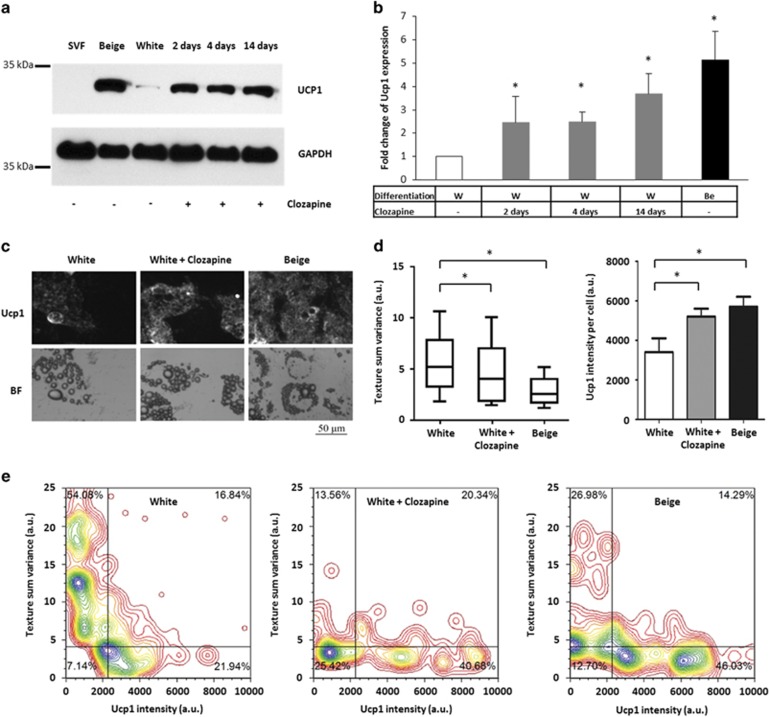
Laser scanning cytometry-based population scale analysis of brown adipogenic differentiation by texture parameters and Ucp1 protein content of *ex vivo*-differentiated single primary adipocytes treated with clozapine. SVF-derived hADMSCs were differentiated and treated as in Figure 1. (**a**) Ucp1 protein expression in one representative adipocyte donor. (**b**) Protein expression level of Ucp1 in the adipocytes of three different SVF donors quantified by densitometry (as compared with untreated white adipocytes). (**c**) Distribution of Ucp1 in clozapine-treated human primary adipocytes. The images were collected with an iCys Research Imaging Cytometer. (**d**) Texture sum variance and Ucp1 protein content of adipocytes per cell. *P<0.05, *n=*3, 2000 cells each donor. (**e**) Dot plot figures of one representative donor based on the identification of browning adipocytes containing small lipid droplets and high levels of Ucp1. hADMSC, human adipose-derived mesenchymal stem cell; SVF, stromal-vascular fraction.

As a next step, we intended to validate the aforementioned effect of clozapine by TaqMan Real-time PCR with reverse transcription. In accordance with our previous results, the expression of *UCP1* was undetectable in hADMSCs and showed a basal level in adipocytes differentiated by the white adipogenic protocol for 14 days.^38^ The differentiating adipocytes were treated with clozapine on the last 2 and 4 days or during the whole white adipogenic differentiation process. We found that *UCP1* was expressed fivefold higher as a result of clozapine administration in each case (Figure 1). A previously described brown adipocyte differentiation protocol,^41^ which turned out to induce a beige program rather than classical brown,^38^ was applied as positive control and resulted in a high expression level of the *UCP1* gene; we are referring to these cells as ‘positive control beige adipocytes'.^38, 41^ We also aimed to analyze the expression of a core set of brown fat-specific genes (*CIDEA*,^52^ *PGC1A*^53^ and *ELOVL3*^54^), an indicator of the mitochondrial enrichment (*CYC1*),^51^ a beige (*TBX1*)^10^ and a classical brown adipocyte marker (*ZIC1*)^6^ in adipocytes differentiated in the presence of clozapine. Significantly elevated expression of *CIDEA*, *CYC1*, *ELOVL3*, *PGC1A* and the beige indicator *TBX1* was found in a concentration-dependent manner compared with the untreated white adipocytes (Figure 1 and Supplementary Figure 2). The long-term clozapine treatment was less effective in the induction of brown and beige-related genes than the above-mentioned browning protocol in most of the cases. The expression of *ZIC1* remained at a low level after clozapine administration excluding that clozapine induces classical brown adipocyte differentiation. Furthermore, clozapine treatment moderately enhanced the expression of beta-oxidation-related mitochondrial genes (*ACOX2*, *CPT1A*, *CPT2* and *ACADM*; Supplementary Figure 3a). In parallel, *PRDM16*^55^ (Figure 1), *CEBPB*^56^ (transcriptional regulators of brown and beige adipocyte development), *CEBPA*, *FABP4*, *LEPTIN* and *PPARG* (general adipocyte markers)^57^ genes expressed at the same level in adipocytes differentiated in the presence of clozapine compared with the untreated ones (Supplementary Figure 4). Undifferentiated progenitors or completely differentiated white or positive control beige adipocytes did not respond to the 12 h-long clozapine treatment (except for the slight upregulation of *TBX1* in differentiated positive control beige adipocytes), suggesting that the adipogenic differentiation program is required for the aforementioned effect of clozapine (Supplementary Figure 5). However, the influence of clozapine on the induction of browning genes was complete in the majority of the cases even if applied only during the last 4 or even 2 days of white adipogenic differentiation (Figure 1). In the collected whole cell lysates of adipocytes that were differentiated in the presence of clozapine, a threefold elevated Ucp1 protein level was detected compared with the untreated cells (Figures 2a and b). Thus, direct clozapine administration during *ex vivo* human white adipocyte differentiation induces a beige-like gene expression pattern and results in the increased amount of Ucp1 protein.

### More hADMSCs and SGBS preadipocytes differentiate into adipocytes with morphological characteristics of browning as a result of clozapine treatment

It has been reported that a bolus clozapine treatment at the early stage of human adipocyte differentiation resulted in an increased lipid content.^58^ We have developed a LSC-based approach to analyze the attached adipocyte cultures at consecutive time points.^40^ This slide-based image cytometry approach combines texture analysis and detection of Ucp1 protein content in single brown adipocytes of mixed cell populations.^38^ In our experiments, up to 50% of the differentiated hADMSCs progressively accumulated lipid droplets; in this respect, there was no difference between clozapine-treated and untreated adipocytes (Figure 2c). However, instead of the formation of large lipid droplets, we found that the long-term clozapine administration resulted in the occurrence of more and smaller droplets in the differentiated adipocytes, similarly but less extensively than in the case of the positive control beige adipocytes. The quantification of this phenomenon by the texture ‘sum variance'^38, 40, 42^ is shown in Figure 2d. Significantly elevated Ucp1 protein content was detected simultaneously in single human adipocytes. Representative images are displayed in Figure 2c and the quantification of Ucp1 content in hADMSC-derived adipocytes of three different donors is presented in Figure 2d. In Figure 2e, we plotted Ucp1 content and texture ‘sum variance' for each differentiated adipocyte. Adipocytes with morphological characteristics of browning were recognized as the ones that contained small lipid droplets and high levels of Ucp1 protein (lower right quadrant of density plot images), in contrast to white adipocytes, which accumulated large lipid droplets and expressed low amount of Ucp1 (upper left quadrant of density plot images).^38^ The biological variance among donors of hADMSCs was summarized in Supplementary Table 1. Using this approach, we demonstrated that depending on individual donors 30–40% of differentiating single human primary adipocytes had the characteristic morphological features of browning cells as a result of clozapine treatment.

**Figure 3:**
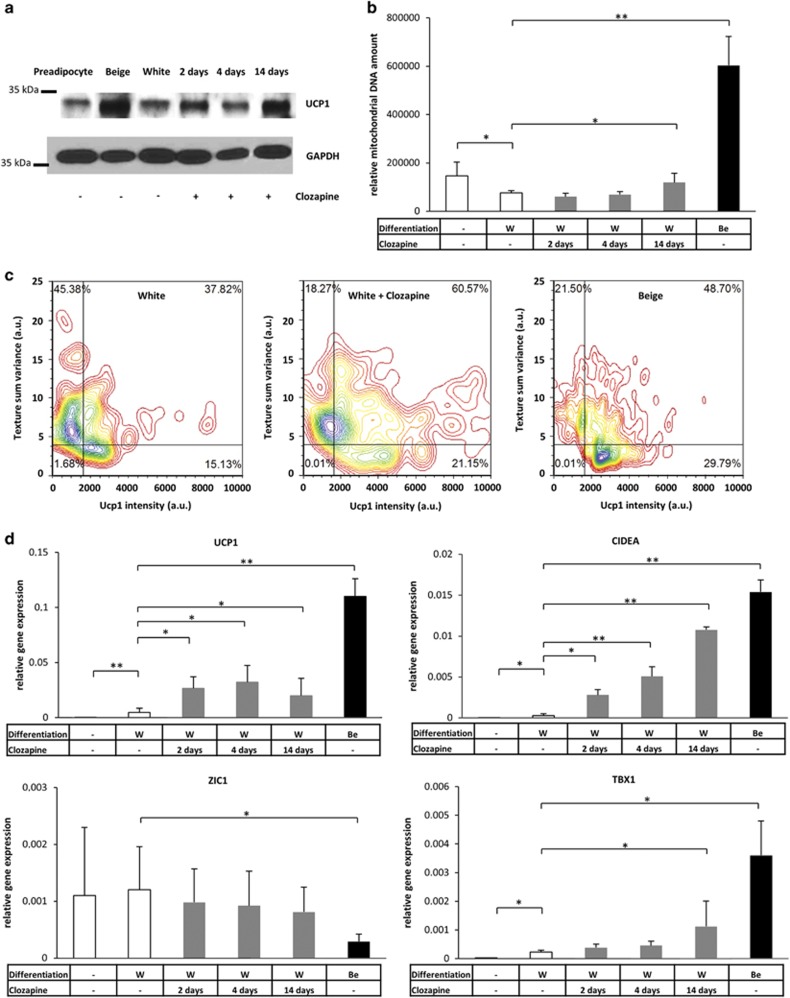
Induction of browning in SGBS human preadipocyte cell line as a result of clozapine treatment during white adipocyte differentiation. Preadipocytes were differentiated for 2 weeks to white (W) or positive control beige (Be) adipocytes. Clozapine (gray bars) was administered at 100 ng ml^−1^ concentration on the last 2 and 4 days or during the whole white adipogenic differentiation process. (**a**) Ucp1 protein expression in one representative SGBS sample. (**b**) Relative mitochondrial DNA amount of SGBS adipocytes determined by qPCR in six different samples. (**c**) Laser scanning cytometry-based population scale analysis of browning in one representative SGBS sample by texture parameters and Ucp1 protein content of adipocytes. (**d**) Relative expression of browning marker genes in SGBS adipocytes. The experiment was repeated six times with samples from independent passages (relative gene expression was determined by RT-qPCR, target genes were normalized to *GAPDH*); *P<0.05, **P<0.01. RT-qPCR, quantitative PCR with reverse transcription; SGBS, Simpson–Golabi–Behmel syndrome.

To exclude the possible impact of heterogeneity and the lower differentiation capacity of the primary cell model, we also investigated the aforementioned effect of clozapine in differentiating SGBS preadipocytes. SGBS cells, which are derived from a patient with SGBS, represent a well-accepted model of human white adipocyte differentiation.^39, 40, 59, 60^ We found elevated Ucp1 protein expression (Figure 3a) and moderately increased mitochondrial DNA amount (Figure 3b) in the cell lysates of SGBS adipocytes that were differentiated in the presence of clozapine compared with the untreated white fat cells. Morphological features of browning (Figure 3c) and a beige-like gene expression pattern (Figure 3d) could be also detected in clozapine-treated SGBS adipocytes. Thus, the data from both primary cells and an adipogenic cell line corroborate that clozapine can shift the differentiation program towards browning.

### Clozapine-treated white adipocytes have increased thermogenic competency but low sensitivity to thermogenic induction *ex vivo*

**Figure 4:**
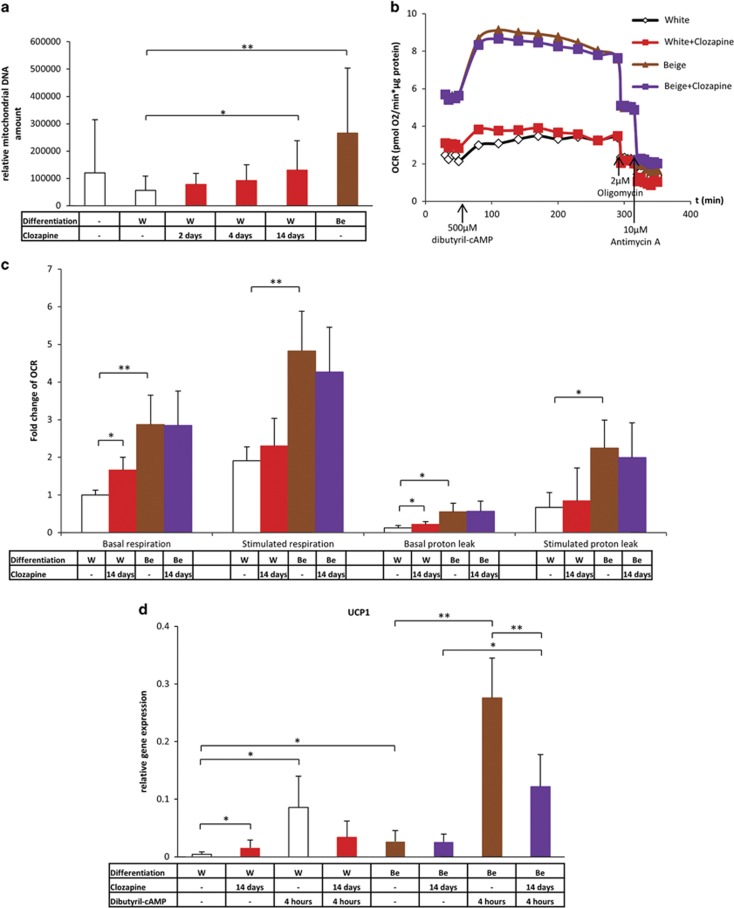
Functional analysis of *ex vivo*-differentiated primary adipocytes treated with clozapine. SVF-derived hADMSCs were differentiated for 2 weeks to white (W) or positive control beige (Be) adipocytes. Clozapine (red bars) was administered at 100 ng ml^−1^ concentration on the last 2 and 4 days or during the whole adipogenic differentiation process. (**a**) Relative mitochondrial DNA amount of human adipocytes determined by quantitative PCR in five different donors. (**b**) Oxygen consumption of SVF-derived adipocytes of one representative donor measured with an XF96 oxymeter. After recording the baseline oxygen consumption, the cells received a single bolus dose of dibutyril-cAMP. Then, stimulated oxygen consumption was recorded at every 30 min. Proton leak respiration was determined after adding oligomycin to block ATP synthase activity. (**c**) Basal, cAMP-stimulated and oligomycin-inhibited oxygen consumption levels (as compared with the basal OCR of white adipocytes) in four different SVF-derived adipocyte donors. (**d**) Effect of short-term cAMP treatment on the expression of *UCP1* gene in primary adipocytes. The experiment was repeated five times with SVFs from independent healthy donors (relative gene expression was determined by RT-qPCR, target gene was normalized to *GAPDH*); *P<0.05, **P<0.01. hADMSC, human adipose-derived mesenchymal stem cell; OCR, oxygen consumption rate; RT-qPCR, quantitative PCR with reverse transcription; SVF, stromal-vascular fraction.

As a next step, we intended to investigate the functional capacity of human primary adipocytes differentiated in the presence of clozapine. Clozapine-treated primary adipocytes had a moderately increased mitochondrial DNA content, to a lesser extent as positive control beige adipocytes (Figure 4a). Figure 4b demonstrates the OC of SVF-derived adipocytes of one representative donor measured by an XF96 oxymeter. The basal mitochondrial respiration of clozapine-treated white adipocytes was moderately elevated as compared with the untreated white cells. In accordance with our previous results,^38^ *ex vivo*-differentiated positive control beige adipocytes had higher basal OCR than the white adipocytes; the presence or absence of clozapine during the differentiation did not affect the basal OCR of positive control beige adipocytes. After the cells received a single bolus dose of cell permeable dibutyril-cAMP mimicking adrenergic stimulation, we found that adipocytes that were differentiated in the presence of clozapine were less capable than the untreated cells to induce their respiration. At the maximal level of induced respiration, the difference in OCR demonstrated at the basal level between clozapine-treated and untreated white adipocytes was abolished. A similar trend was observed when cells were treated with oligomycin that blocks the activity of ATP synthase and provides information on proton leak respiration (Figure 4c). Considering the fact that dibutyril-cAMP enhanced lipolysis might increase the release of fatty acids by adipocytes, which can stimulate respiration by artefactual mitochondrial uncoupling,^44^ we carried out OC measurements including 2% BSA in the respiration buffer. Although we observed weaker induction of OCR in response to dibutyril-cAMP in each differentiated sample, the aforementioned effects of clozapine on the metabolic parameters of adipocytes could be detected in this experimental condition as well (Supplementary Figure 6).

Recent findings revealed a substrate cycle of creatine metabolism, which enhances energy expenditure in the mitochondria of beige adipocytes. Mitochondrial creatine kinase 1 or 2 catalyzes the conversion of creatine and utilizes ATP to create phosphocreatine (PCr) and ADP, before Phospho1 liberates the high-energy phosphate group from PCr.^45^ When adipocytes differentiated in the presence of clozapine were treated with etomoxir (O-carnitine palmitoyltransferase-1 inhibitor) or β-guanidinopropionic acid (creatine analog which reduces creatine levels in the cells), the basal and cAMP-stimulated OCRs were decreased at a higher extent then in the case of white adipocytes. These data demonstrate that the clozapine-induced browning cells (similar to positive control beige adipocytes) utilize more fatty acids by beta-oxidation than white adipocytes and further increase their energy expenditure by activating the recently identified futile cycle of creatine metabolism (Supplementary Figures 3b and 7).

In parallel, we analyzed the changes in the gene expression of the previously shown brown and beige adipocyte markers as a result of a 4 h-long dibutyril-cAMP treatment that serves as an *ex vivo* model of thermogenic induction.^6^ In line with the previously described results, white adipocytes expressed *UCP1* gene at a basal level. White adipocytes differentiated in the presence of clozapine had a significantly elevated *UCP1* expression compared with the untreated cells. Positive control beige adipocytes expressed *UCP1* mRNA at a higher level; in this case the clozapine treatment did not affect *UCP1* expression. A similar trend was observed in the case of *PGC1A*. The *UCP1* and *PGC1A* expression showed a robust (ten- and fivefold, respectively) upregulation in response to cAMP treatment of white and positive control beige adipocytes differentiated in the absence of clozapine. This effect of thermogenic induction was less manifested in clozapine-generated beige adipocytes, with regard to *UCP1*. The responsiveness of positive control beige adipocytes differentiated in the presence of clozapine was also reduced (Figure 4d and Supplementary Figure 8). The expression of *CYC1*, *CIDEA*, *ELOVL3*, *PRDM16*, *TBX1* and *ZIC1* did not change significantly as a result of thermogenic induction in either clozapine-treated or untreated adipocytes (data not shown). Of note, beta-oxidation and creatine-driven substrate cycle was enhanced by cAMP in a similar extent in clozapine-induced as in positive control beige adipocytes (Supplementary Figures 3b and 7). Although the demonstrated gene expression, morphological and functional data suggest that clozapine treatment induces the thermogenic competency of differentiating white adipocytes, these ‘masked' beige cells are less capable to respond to β-adrenergic induction of thermogenesis.

### Clozapine induces browning via inhibiting 5HT-receptor-mediated signaling

Next, we intended to learn the molecular mechanism, which can explain the browning effect of clozapine. Although initially introduced as a dopamine receptor antagonist in the clinical practice, clozapine has a very diverse molecular interaction profile. It binds to different 5HT, muscarinic and histamine receptors and antagonizes adrenergic alpha 1 and 2 or various dopamine receptors.^61, 62, 63^ Fourteen different 5HT-receptor types have been already described^64^ and most of them are expressed by white^65, 66^ and brown adipocytes^67, 68^ in rodents. In human adipocytes, the expression of *HTR1D*, *HTR2A*, *HTR3A* and *HTR7* were reported in different studies.^12, 69^

**Figure 5:**
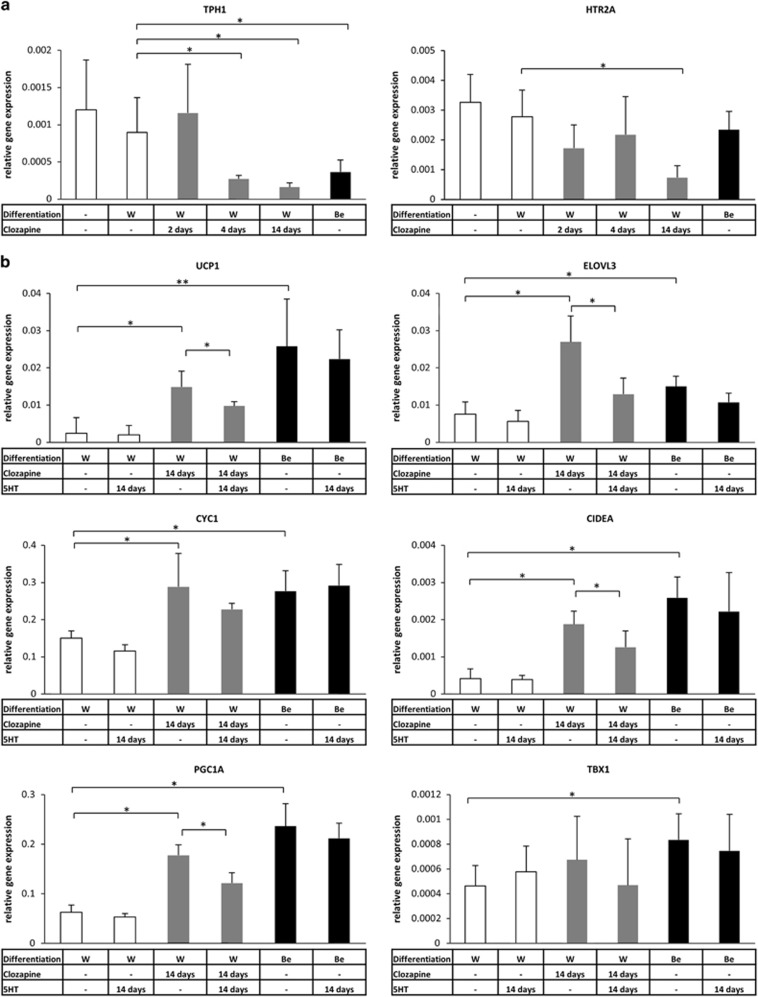
Effect of 5HT on the induction of browning in *ex vivo*-differentiated primary human adipocytes treated with clozapine. SVF was differentiated for 2 weeks to white (W) or positive control beige (Be) adipocytes. 100 ng ml^−1^ clozapine (gray bars) and/or 10 μm 5HT was administered on the last 2 and 4 days or during the whole adipogenic differentiation process. (**a**) Relative expression of Tryptophan Hydroxylase 1 and *HTR2A* receptor in adipocytes in response to clozapine administration. (**b**) Relative expression of browning marker genes as a result of clozapine and 5HT treatment. The experiment was repeated five times with SVFs from independent healthy donors (Relative gene expression was determined by RT-qPCR, target genes were normalized to *GAPDH*); *P<0.05, **P<0.01. 5HT, serotonin; RT-qPCR, quantitative PCR with reverse transcription; SVF, stromal-vascular fraction.

Primarily, we investigated the expression of the above-mentioned 5HT-receptor genes. In our experiments, we found that *HTR2A* was expressed at the highest level in hADMSCs and in differentiated adipocytes. Interestingly, long-term clozapine administration resulted in the downregulation of *HTR2A* gene (Figure 5a). In addition, the *HTR1D* and *HTR7* genes were expressed at a lower extent. In contrast to *HTR2A*, their expression was not altered in response to clozapine treatment (Supplementary Figure 9). We found a similar expression pattern in SGBS adipocytes, except for the undetectable expression level of *HTR7* (data not shown). The rate-limiting step of 5HT synthesis in peripheral tissues is catalyzed by Tryptophan Hydroxylase 1 (*TPH1*).^70^ The expression of *TPH1* was detectable in hADMSCs (Figure 5a) and in SGBS preadipocytes (data not shown), and did not change as a result of white adipocyte differentiation, suggesting that these cells are capable of autonomously generating and releasing 5HT during adipogenesis. However, we found decreased *TPH1* expression in clozapine-induced and in positive control beige adipocytes (Figure 5a). As a next step, exogenous 5HT was administered daily to the differentiating primary adipocytes. We found that the upregulation of the majority of the browning markers was significantly attenuated when clozapine-induced beige adipocytes were differentiated in the presence of 5HT (Figure 5b). The expression of white and general adipogenic markers was not changed as a result of 5HT administration (Supplementary Figure 10). The SGBS adipocytes differentiated in the presence of clozapine similarly responded to the 5HT treatment (data not shown). Of note, the 5HT administration alone did not modify the gene expression pattern of differentiating white and positive control beige adipocytes. Our results suggest that disturbance of 5HT production or 5HT-mediated signaling might, at least partially, explain the browning effect of clozapine.

## Discussion

The mechanisms underlying SGA-induced metabolic dysfunctions remain barely understood.^26^ Only a few studies investigated the direct effect of SGAs on differentiating human adipocytes.^47, 58^ To fill this hiatus and to follow up our previous study where we looked at selected adipogenic, cell cycle-related and pro-inflammatory genes in SGA-treated human white adipocytes,^35^ in the present study we found that clozapine administration unexpectedly resulted in the upregulation of *UCP1* (Supplementary Figure 1). This prompted us to examine how the propensity of hADMSCs and SGBS preadipocytes to differentiate into heat-generating brown or beige cells is influenced by clozapine. The administration of the drug at 20-fold higher concentration than the upper limit of therapeutic reference range resulted in a significant inhibition of the differentiation of a murine brown preadipocyte cell line.^71^ However, toxic effects of the treatment for the cultured cells cannot be excluded at this high clozapine concentration.^47^ On the other hand, clozapine administration at the concentrations of therapeutic reference range had a slight positive effect on the expression of the selected BAT marker genes in an *in vitro* mouse model.^71^ In our experiments, clozapine treatment resulted in significantly elevated expression of several brown marker genes, including the beige indicator *TBX1*, in differentiating human adipocytes.

We complemented the measurements of gene expression changes from total cell lysates with the recently described LSC approach that helped us to specifically identify browning cells in mixed adipocyte populations.^38^ When the adipocytes were differentiated in the presence of clozapine for two weeks, the rate of browning cells increased by approximately 1.5-fold. Clozapine did not, however, alter the gene expression pattern of fully differentiated white or positive control beige adipocytes. This indicates that clozapine, most probably, induces the commitment of mesenchymal adipocyte progenitor cells to beige adipocytes at some point during the differentiation. The ratio of energy-dissipating beige and energy-storing white adipocytes (which fundamentally defines the thermogenic competency of each individual) is determined, at least partially, during early differentiation of mesenchymal progenitors into adipocyte subtypes.^14, 16, 17^ The upregulation of the marker genes of browning was nearly the same when the administration of clozapine took place only on the last 2 or 4 days of the white adipogenic differentiation suggesting that clozapine is able to induce this developmental shift from white to beige even at the later stage of the *ex vivo* differentiation process.

Recently, two groups reported independently that peripheral 5HT reduced the beige potential and the sensitivity of brown and beige adipocytes to thermogenic induction in a cell autonomous manner in mice.^43, 70^ Increased levels of peripheral 5HT^72^ and polymorphisms in the *TPH1* gene^73^ are associated with obesity. We found that *TPH1*, the enzyme which catalyzes the rate-limiting step of 5HT production outside the central nervous system, and 5HT-receptor genes are expressed in human primary and SGBS adipocytes and by undifferentiated precursors. Furthermore, exogenous 5HT partially abrogated the upregulation of browning marker genes that were induced by clozapine administration. In our experiments, out of the 5HT receptors, *HTR2A* was expressed at the highest level. *HTR2A* initiates Gq signaling, which was recently reported to abolish browning in mice and in human adipocytes. In parallel, the expression of Gq protein correlated inversely with *UCP1* in WAT *in vivo*.^74^ As clozapine is able to antagonize several 5HT receptors, with the highest affinity to *HTR2A*;^62^ the disturbance of 5HT-receptor-mediated Gq signaling might, at least partially, explain the unexpected phenomenon described in the present study assuming that 5HT or similarly acting compounds are generated and released from cells during white adipocyte differentiation.

Previously, it was found that clozapine enhances the accumulation of lipids in differentiating human adipocytes which could stand in line with its documented effect to promote obesity.^58^ However, we failed to detect more or larger lipid droplets in the clozapine-treated adipocytes by LSC. These cells had smaller lipid droplets in multilocular arrangement, more mitochondrial DNA and expressed more Ucp1 protein than the untreated white adipocytes. On the other hand, it should be emphasized that beige fat cells differentiated from precursors (which process is enhanced by clozapine administration) do not activate the effective thermogenic program unless they are stimulated with certain inducers, for example β-adrenergic agonists.^7, 10, 75^ The question may arise why *in vivo* administration of clozapine leads to fat gain and obesity. A similar discrepancy has been observed with thiazolidinediones that act through the nuclear receptor PPARG.^76, 77^ Clinical trials implicated that rosiglitazone increases the risk of obesity, congestive heart failure and myocardial infarction in patients who received the drug for type 2 diabetes mellitus,^78, 79, 80^ while it was capable of increasing the thermogenic competency of WAT in mice^7^ and inducing browning of human adipocytes *ex vivo.*^41^ Our data suggest that adipocytes differentiated in the presence of clozapine are less sensitive to cAMP stimulation. In line with this, altered cAMP signaling has been reported in the brain as a result of SGA treatment in rodents^81, 82^ and in humans.^83^ The observed incomplete thermogenic response of clozapine-generated beige cells to β-adrenergic agonist can be one of the reasons why these ‘masked' beige adipocytes function ineffectively also *in vivo*. Results from *in vivo* studies focusing on the effect of SGAs on the sympathetic nervous system have been conflicting.^84, 85, 86, 87^ The β3-adrenergic pathway which has a key role in the activation of thermogenesis in brown and beige adipocytes^20, 88, 89^ has never, to our knowledge, been investigated in this regard. Further investigations will help to identify the ‘masked' beige cells differentiated in response to clozapine treatment *in vivo* and to find ways to induce their thermogenic function independently from the sympathetic nervous system.
