# Supplementary material for: Clozapine modifies the differentiation program of human adipocytes inducing browning
Source: Transl Psychiatry. 2016 Nov 29;6(11):e963–. doi: 10.1038/tp.2016.230 (PMC5290354; doi:10.1038/tp.2016.230)
Supplement: Supplementary Information [file tp2016230x1.pdf]

Supplementary Information

Clozapine modifies the differentiation program of human adipocytes inducing browning

Endre Kristóf, Quang-Minh Doan-Xuan, Anitta Kinga Sárvári, Ágnes Klusóczki, Pamela Fischer-Posovszky, Martin Wabitsch, Zsolt Bacso, Péter Bai, Zoltán Balajthy & László Fésüs*

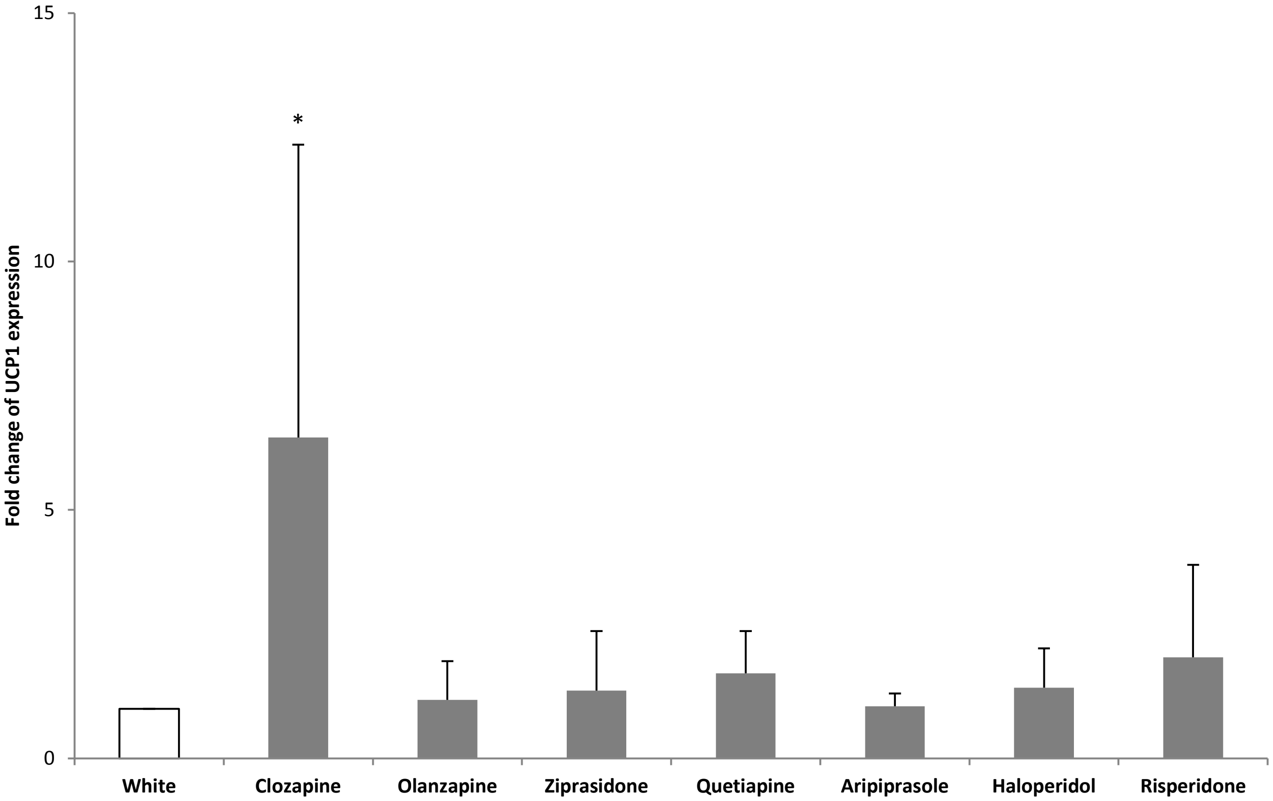

Supplementary Figure 1. UCP1 gene expression (as compared to untreated white adipocytes) in differentiated human primary adipocytes in response to antipsychotics treatment. Confluent SVF derived hADMSCs were differentiated to adipocytes *ex vivo* in the presence of antipsychotics. The experiment was repeated four times with SVFs from independent healthy donors; *p=0.0409.

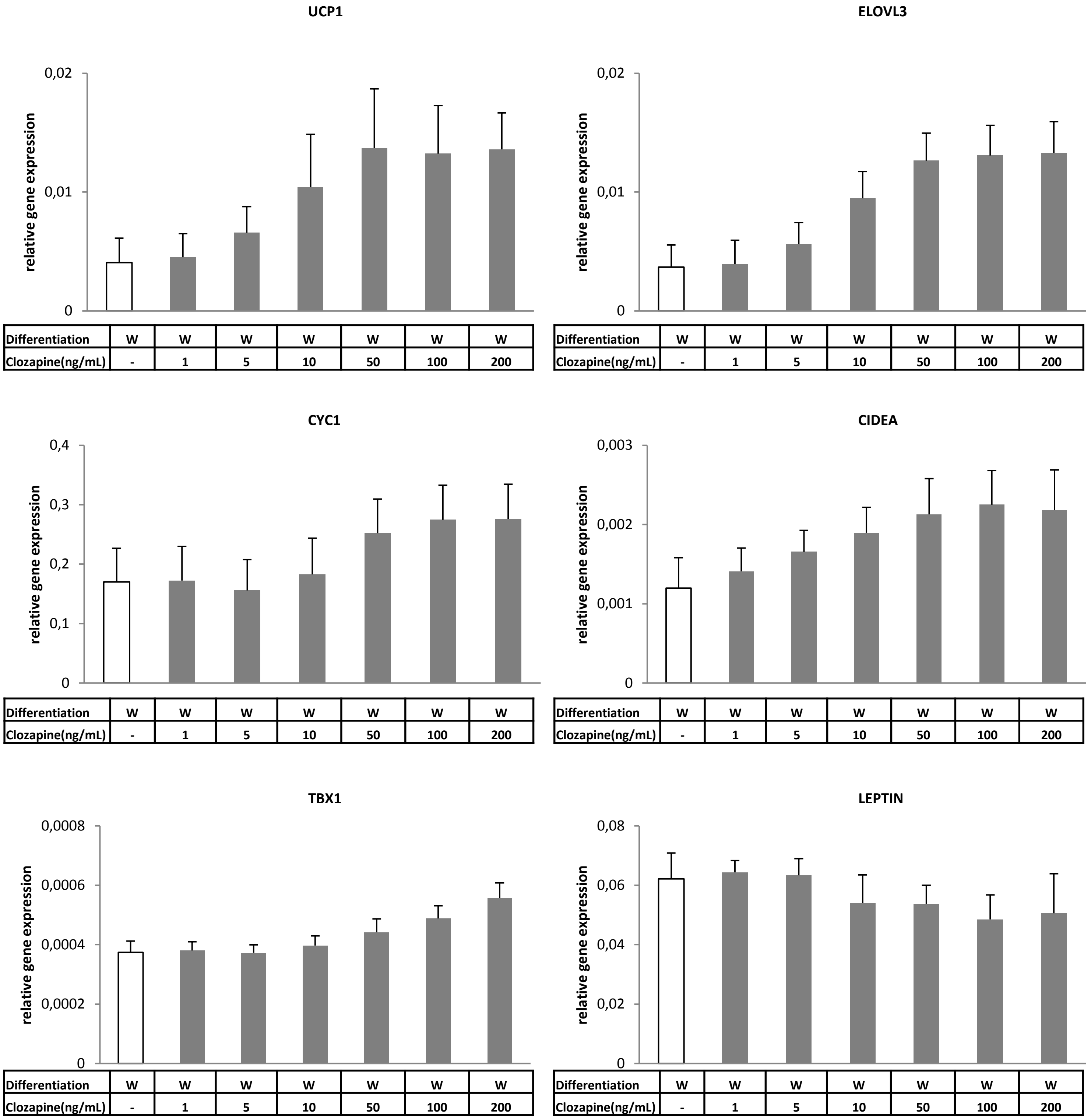

Supplementary Figure 2. Dose dependence of clozapine treatment during *ex vivo* white adipocyte differentiation on the relative expression of browning and adipogenic marker genes in primary human adipocytes. SVF was differentiated for two weeks to white (W) adipocytes. 1-200 ng/mL clozapine (grey bars) was administered during the whole white adipogenic differentiation process. The experiment was repeated three times with SVFs from independent healthy donors (Target genes were normalized to GAPDH).

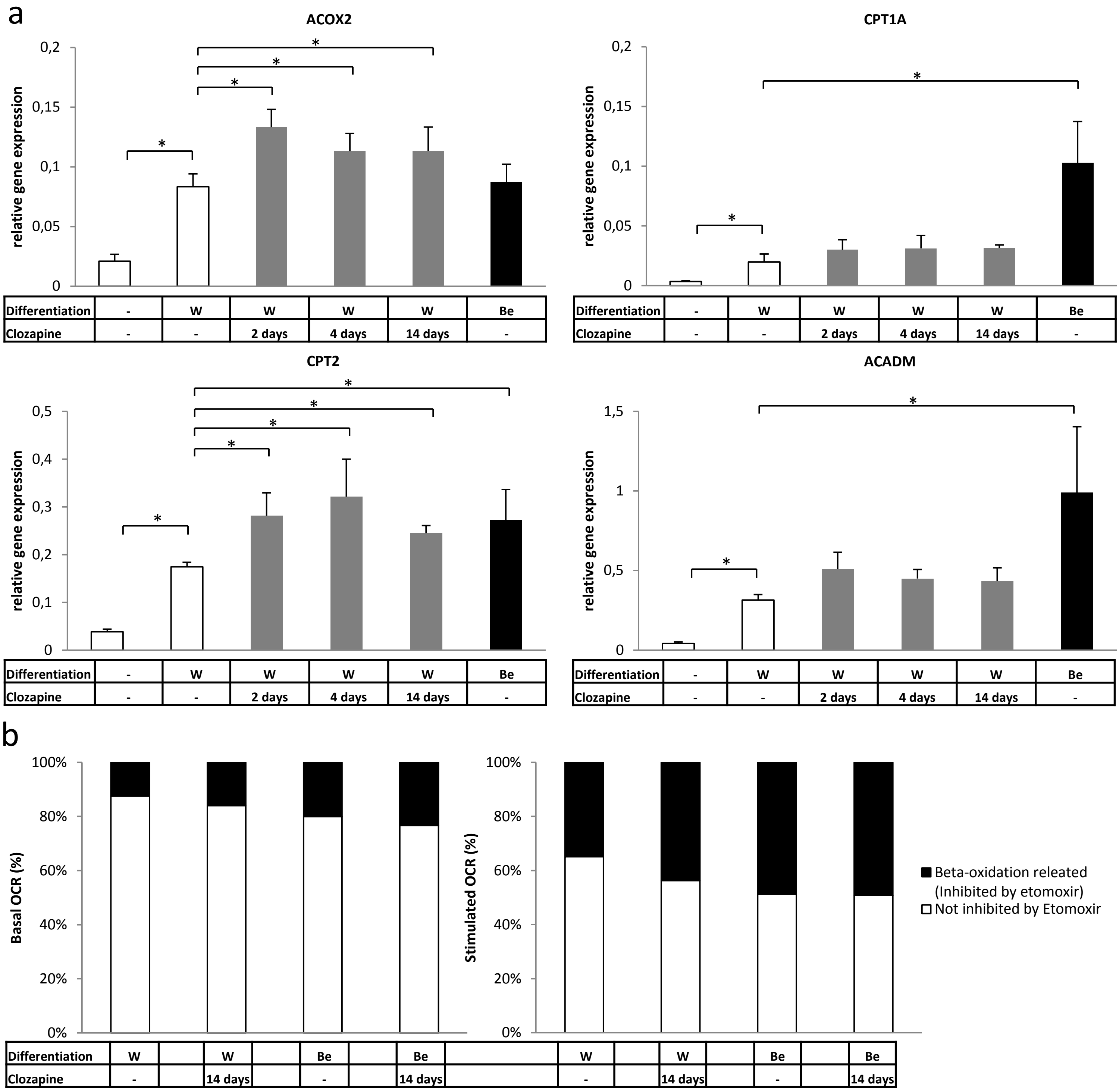

Supplementary Figure 3. Involvement of beta-oxidation in the metabolism of *ex vivo* differentiated primary adipocytes treated with clozapine. SVF was differentiated for two weeks to white (W) or positive control beige (Be) adipocytes. 100 ng/mL clozapine was administered on the last 2 and 4 days or during the whole adipogenic differentiation process. The experiment was repeated four times with SVFs from independent healthy donors **(a)** Relative expression of beta-oxidation related genes (Target genes were normalized to GAPDH); *$p<0.05$. **(b)** Etomoxir inhibited (5 μM final concentration) relative oxygen consumption levels (as compared to basal and cAMP stimulated OCR of each sample) measured by an XF96 oxymeter.

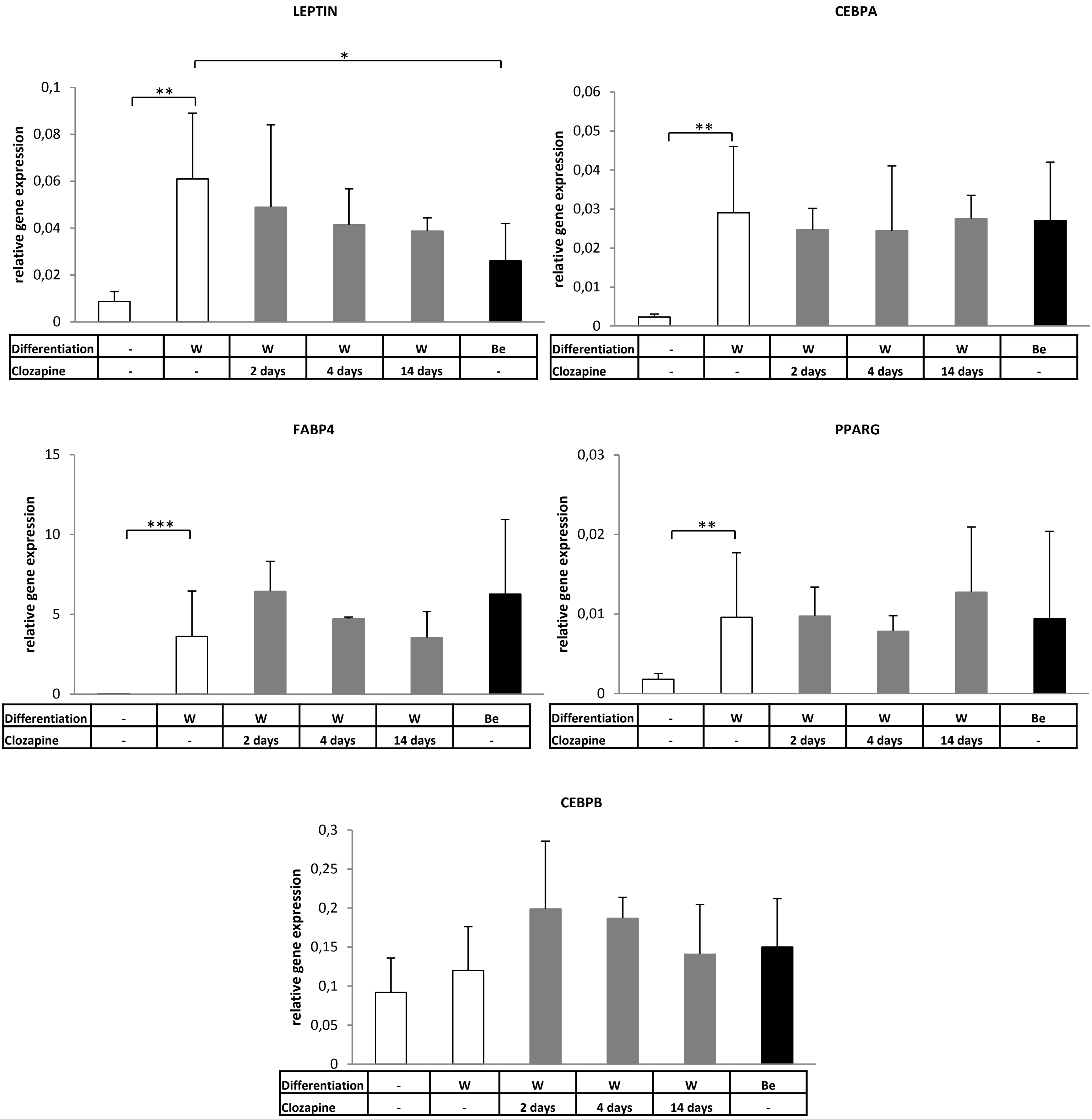

Supplementary Figure 4. Relative expression of white, brown and general adipogenic marker genes in primary human adipocytes as a result of clozapine treatment during *ex vivo* white or beige adipocyte differentiation. SVF was differentiated for two weeks to white (W) or positive control beige (Be) adipocytes. 100 ng/mL clozapine (grey bars) was administered on the last 2 and 4 days or during the whole white adipogenic differentiation process. The experiment was repeated six times with SVFs from independent healthy donors (Target genes were normalized to GAPDH); *p<0.05, **p<0.01, ***p<0.001.

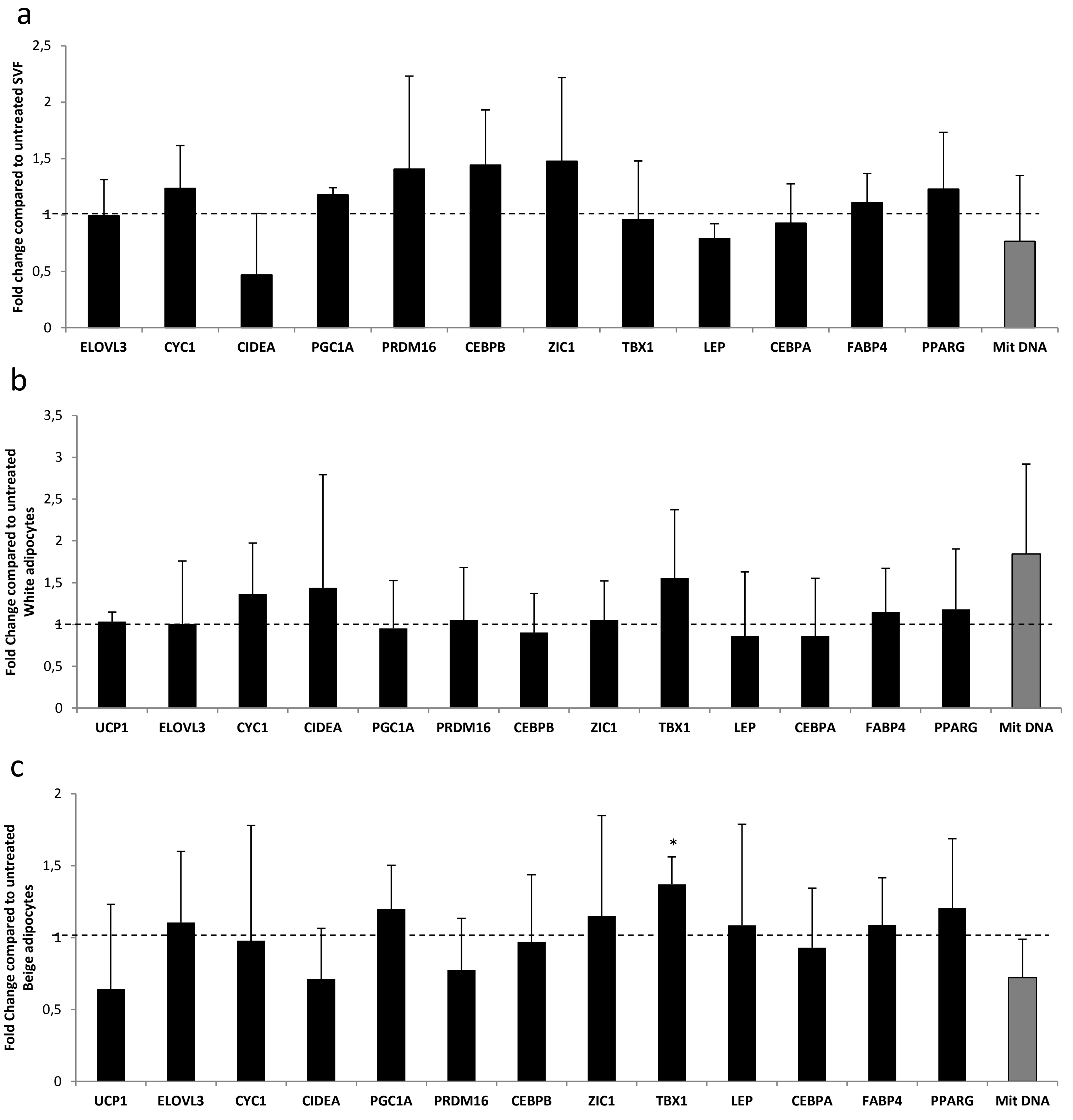

Supplementary Figure 5. Effect of short-term clozapine treatment on the expression of selected adipocyte marker genes and mitochondrial DNA amount in primary human SVF, white and beige adipocytes (as compared to untreated cells). 100 ng/mL clozapine was administered for 12 hours to undifferentiated SVF **(a)** or to fully differentiated white **(b)** or positive control beige **(c)** adipocytes. The experiment was repeated five times with SVFs from independent healthy donors. *$p<0.05$ (The expression of UCP1 could not be detected in SVF).

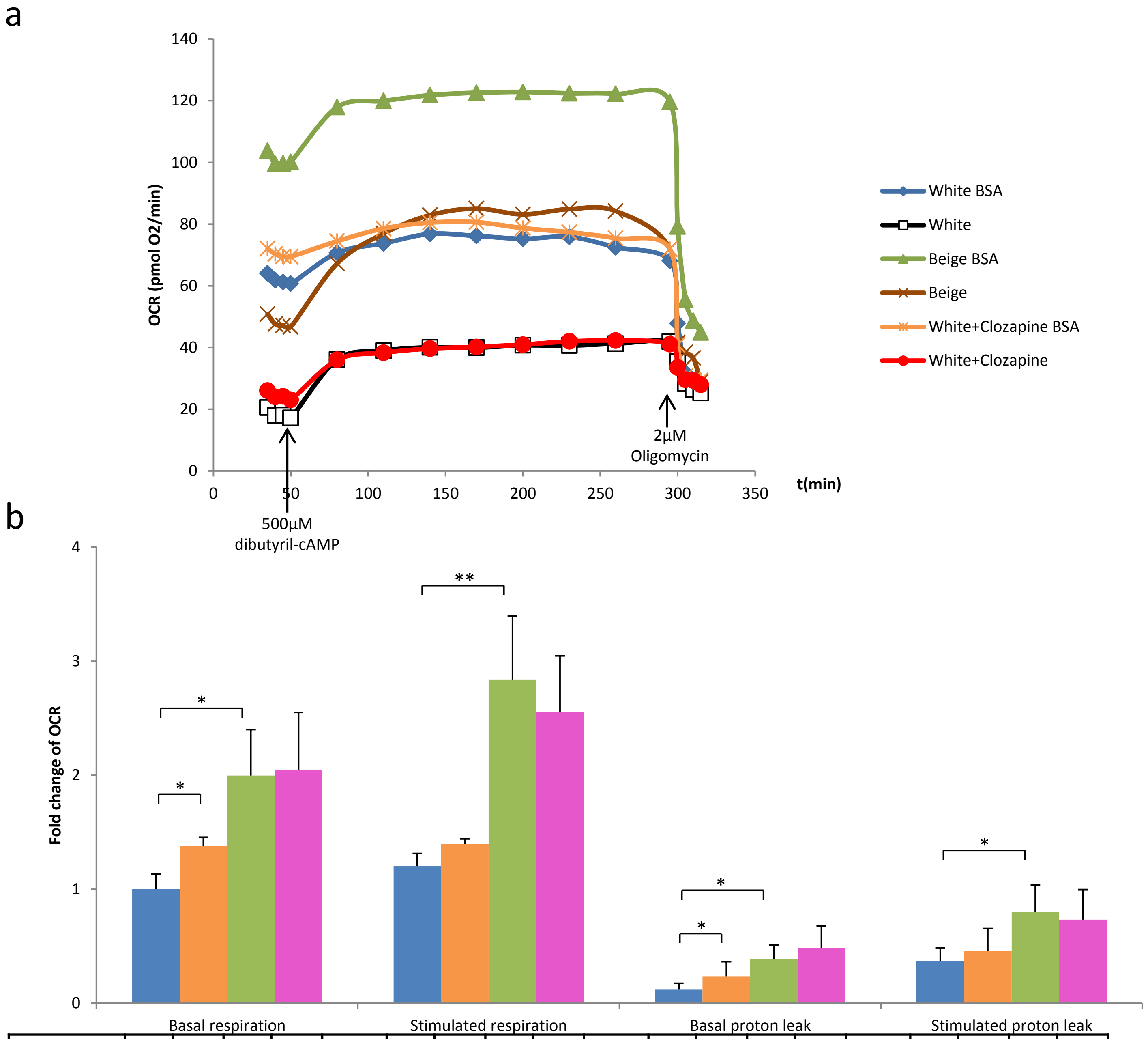

Differentiation	W	W	Be	Be		W	W	Be	Be		W	W	Be	Be		W	W	Be	Be
Clozapine	-	14 days	-	14 days		-	14 days	-	14 days		-	14 days	-	14 days		-	14 days	-	14 days

Supplementary Figure 6. Effect of blocking fatty acid release by bovine serum albumin (BSA) on the respiration of *ex vivo* differentiated primary adipocytes treated with clozapine. SVF was differentiated for two weeks to white (W) or positive control beige (Be) adipocytes. 100 ng/mL clozapine was administered during the whole adipogenic differentiation process. **(a)** Oxygen consumption of SVF derived adipocytes of one representative donor measured by an XF96 oxymeter. After recording the baseline oxygen consumption, cells received a single bolus dose of dibutyril-cAMP modelling adrenergic stimulation. Then, stimulated oxygen consumption was recorded at every 30 min. Proton leak respiration was determined after adding 2 µM oligomycin to block ATP synthase activity. **(b)** Basal, cAMP stimulated and oligomycin inhibited oxygen consumption levels (as compared to basal OCR of white adipocytes). The experiment was repeated four times with SVFs from independent healthy donors. The respiration buffer contained 2% BSA.*p<0.05, **p<0.01.

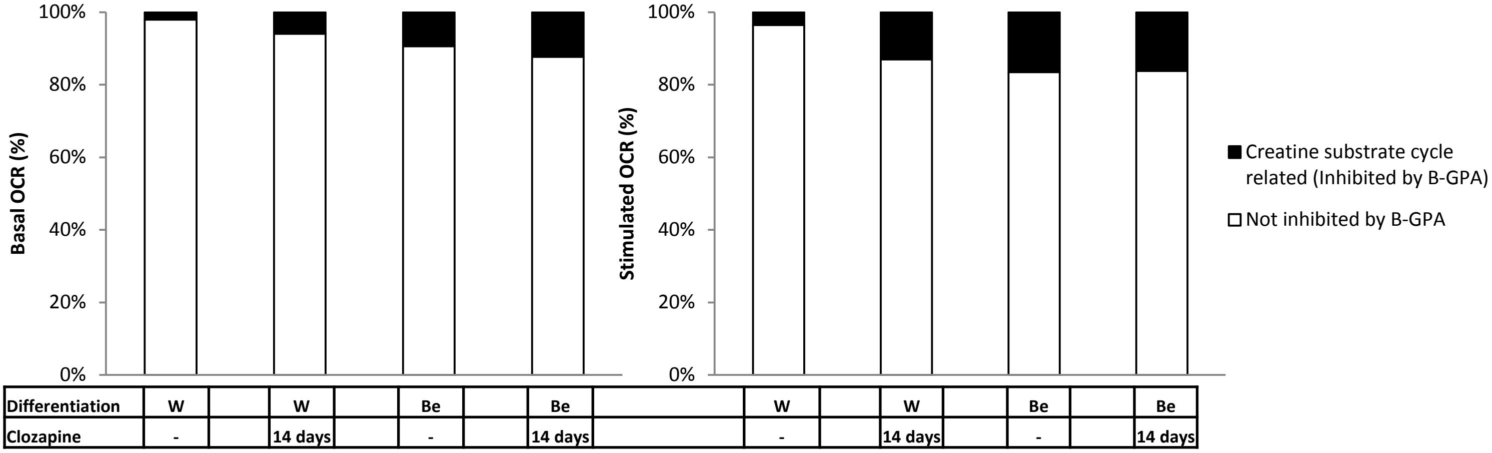

Supplementary Figure 7. Involvement of the creatine-driven futile cycle in the metabolism of *ex vivo* differentiated primary adipocytes treated with clozapine. β-guanidinopropionic acid (β-GPA) inhibited (2 mM final concentration) relative oxygen consumption levels (as compared to basal and cAMP stimulated OCR of each sample) measured by an XF96 oxymeter. SVF was differentiated for two weeks to white (W) or positive control beige (Be) adipocytes. 100 ng/mL clozapine was administered during the whole adipogenic differentiation process. The experiment was repeated four times with SVFs from independent healthy donors.

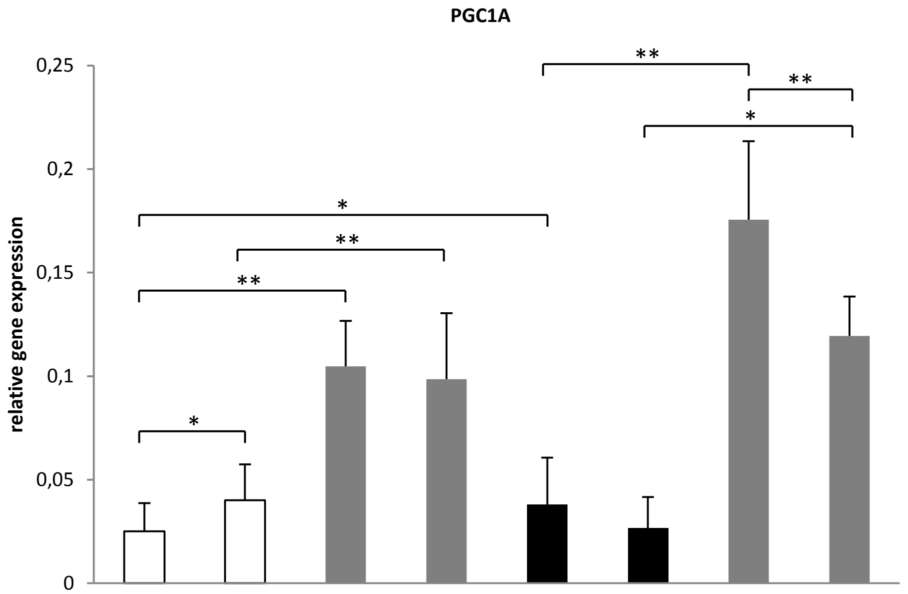

Differentiation	W	W	W	W	Be	Be	Be	Be
Clozapine	-	14 days	-	14 days	-	14 days	-	14 days
Dibutyril-cAMP	-	-	4 hours	4 hours	-	-	4 hours	4 hours

Supplementary Figure 8. Effect of short-term cAMP treatment on the expression of PGC1A gene in primary human adipocytes that had been treated with clozapine during *ex vivo* white or beige adipocyte differentiation. SVF derived hADMSCs were differentiated for two weeks to white (W) or positive control beige (Be) adipocytes. 100 ng/mL clozapine was administered during the whole adipogenic differentiation process. Then cells received a single bolus dose of 500 µM dibutyril-cAMP (grey bars) modelling adrenergic stimulation. The experiment was repeated five times with SVFs from independent healthy donors (Target genes were normalized to GAPDH); *p<0.05, **p<0.01.

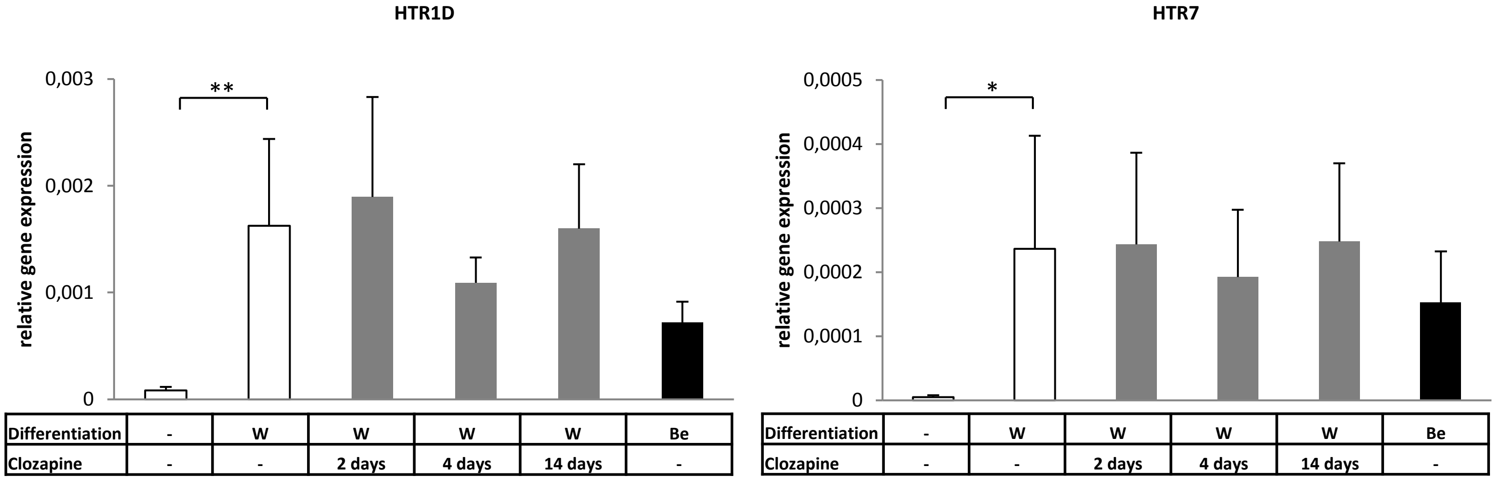

Supplementary Figure 9. Relative expression of 5HT receptors in primary human adipocytes as a result of clozapine treatment during *ex vivo* white or beige adipocyte differentiation. SVF was differentiated for two weeks to white (W) or positive control beige (Be) adipocytes. 100 ng/mL clozapine (grey bars) was administered on the last 2 and 4 days or during the whole white adipogenic differentiation process. The experiment was repeated five times with SVFs from independent healthy donors (Target genes were normalized to GAPDH); *p<0.05, **p<0.01.

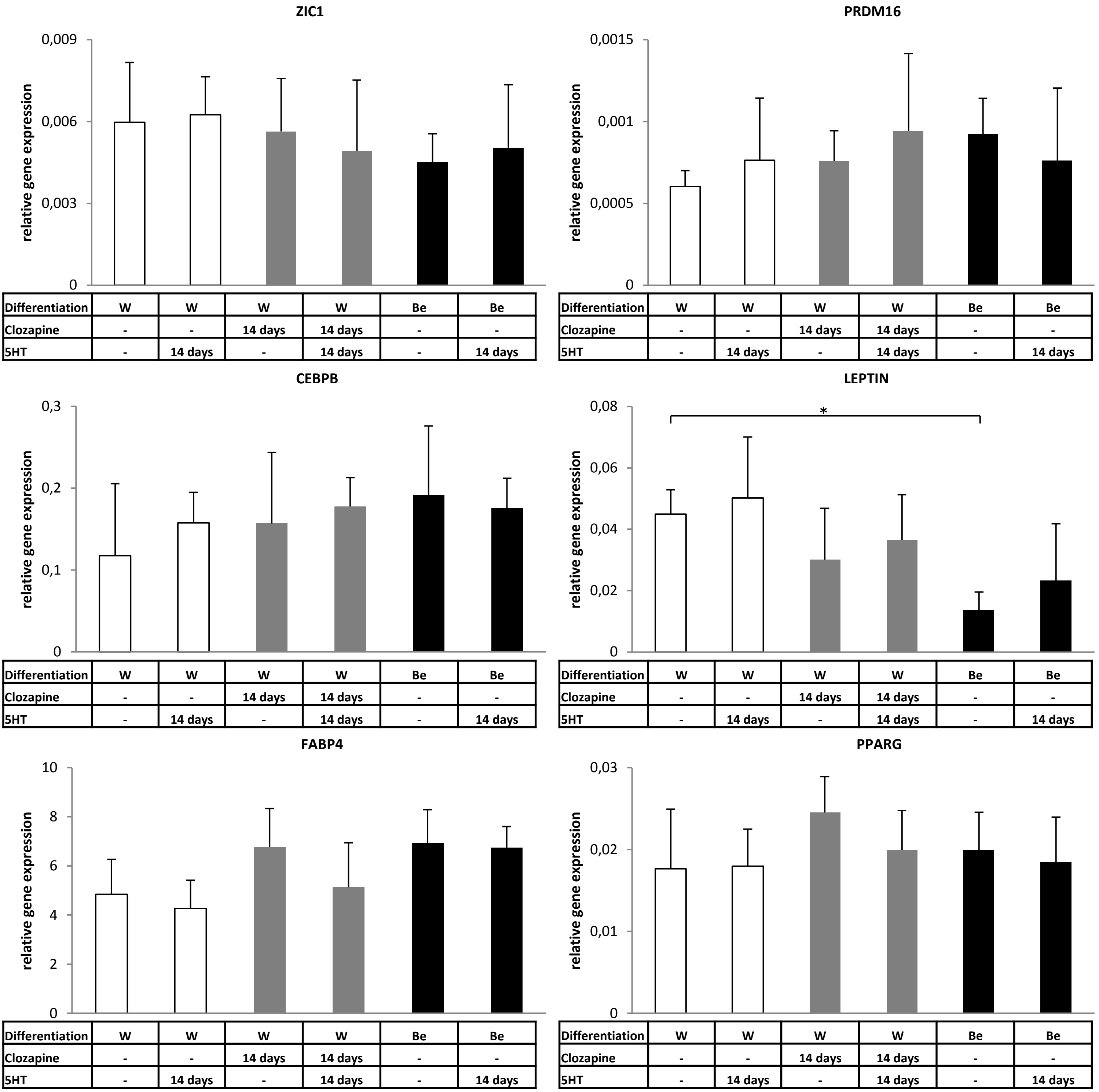

Supplementary Figure 10. Relative expression of white, brown and general adipogenic marker genes in primary human adipocytes as a result of clozapine and 5HT treatment during *ex vivo* white or beige adipocyte differentiation. SVF was differentiated for two weeks to white (W) or positive control beige (Be) adipocytes. 100 ng/mL clozapine (grey bars) and/or 10 μM 5HT were administered during the whole adipogenic differentiation process. The experiment was repeated five times with SVFs from independent healthy donors (Target genes were normalized to GAPDH); *p<0.05.

Donors	White differentiation protocol		Beige differentiation protocol		White differentiation protocol + Clozapine	
	% of differentiated adipocytes					
	Texture↑ Ucp1↓	Texture↓ Ucp1↑	Texture↑ Ucp1↓	Texture↓ Ucp1↑	Texture↑ Ucp1↓	Texture↓ Ucp1↑
**1**	42.34	29.20	26.98	46.03	13.56	40.66
**2**	56.64	17.60	8.60	44.09	14.55	25.45
**3**	54.08	21.94	8.62	58.62	28.89	33.23

Supplementary Table 1. Laser Scanning Cytometry based population scale analysis of clozapine effect on *ex vivo* adipogenic differentiation by texture parameters and Ucp1 protein content of primary adipocytes showing the biological variance of different donors. Browning adipocytes are identified as containing small lipid droplets (Texture↓) and high level of Ucp1 protein (Ucp1↑).
